# Discovery and Treatment of Action Potential‐Independent Myotonia in Hyperkalemic Periodic Paralysis

**DOI:** 10.1002/acn3.70134

**Published:** 2025-07-14

**Authors:** Chris Dupont, Adam Deardorff, Murad Nawaz, Andrew A. Voss, Mark M. Rich

**Affiliations:** ^1^ Department of Neuroscience, Cell Biology and Physiology Wright State University Dayton Ohio USA; ^2^ Department of Clinical Neurosciences Wright State University Dayton Ohio USA; ^3^ Department of Biological Sciences Wright State University Dayton Ohio USA

**Keywords:** action potential, excitation, skeletal muscle, sodium channel, stiffness

## Abstract

**Objective:**

Hyperkalemic periodic paralysis (hyperKPP) is characterized by attacks of transient weakness. A subset of hyperKPP patients suffers from transient involuntary contraction of muscle (myotonia). The goal of this study was to determine mechanisms causing myotonia in hyperKPP.

**Methods:**

Intracellular electrophysiology, single‐fiber Ca^2+^ imaging, and whole muscle contractility studies were performed in a mouse model of hyperKPP.

**Results:**

Myotonia in hyperkPP was caused by both involuntary myogenic action potentials (AP myotonia) lasting less than 5 min and action potential‐independent myotonia (non‐AP myotonia) lasting over 1 h. Non‐AP myotonia was caused by prolonged subthreshold depolarization and elevated intracellular Ca^2+^ in the absence of action potentials. Treatment with dantrolene effectively mitigated non‐AP myotonia, suggesting that the source of Ca^2+^ was the sarcoplasmic reticulum. Although non‐AP myotonia occurred in the absence of action potentials, Na^+^ channel blockers were effective as therapy.

**Discussion:**

We propose myotonia in hyperKPP occurs via two mechanisms: (1) suprathreshold depolarization triggering action potentials that are detectable with EMG and (2) sustained subthreshold depolarization resulting in Na^+^ overload and Ca^2+^ leak from the sarcoplasmic reticulum. Notably, clinical diagnostics such as EMG cannot detect the second mechanism as it occurs in the absence of action potentials. Currently, only a minority of patients with hyperKPP are treated with Na^+^ channel blockers and none are treated with dantrolene. Our data suggest hyperKPP patients, as well as patients with a number of other neuromuscular disorders, may benefit from trials of these therapies, even if they do not have myotonia detectable clinically or by EMG.

## 1 Introduction

Hyperkalemic periodic paralysis (hyperKPP) is a dominantly inherited muscle channelopathy caused by gain‐of‐function mutations of the Nav1.4 sodium channel (SCN4A). The mutations increase the percentage of channels in a mode lacking inactivation (persistent Na^+^ current or NaP) [1, 2, 3]. Work with heterologous expression systems suggests NaP in hyperKPP causes weakness by depolarizing the muscle enough to induce loss of the ability to fire action potentials [3, 4, 5, 6]. However, no voltage clamp recordings have been performed to demonstrate an increase in NaP in muscle from the mouse model of hyperKPP.

While hyperKPP is primarily known for causing transient attacks of muscle weakness associated with elevated serum K^+^, approximately 50% of patients also suffer from involuntary muscle contraction (myotonia) [5, 6, 7], which can be painful and debilitating and has been comparatively understudied. These contractions are thought to be due to involuntary firing of myogenic action potentials triggered by suprathreshold depolarizations (myotonic discharges), which are readily detected by EMG [5, 6, 7]. Yet, there is little data exploring the mechanistic underpinnings of these action potentials or, in fact, the contractions themselves. Using a combination of intracellular electrophysiology, single‐fiber Ca^2+^ imaging, and whole muscle contractility studies, we sought to better elucidate the cellular mechanisms underlying myotonia and its treatment in a mouse model of hyperKPP.

We began our studies of a mouse model of hyperKPP by demonstrating via voltage clamp that NaP is, indeed, increased in hyperKPP muscle. We next identified two distinct mechanisms of myotonia, both of which are triggered by NaP. The first is a suprathreshold depolarization of the muscle that triggers a waxing and waning of action potentials that is independent of nerve stimulation. This is the archetypical mechanism for triggering myotonia and lasts a few minutes. The second, novel mechanism, is due to elevation of intracellular Ca^2+^ in fibers not firing action potentials. This mechanism is responsible for myotonia lasting more than an hour. Both mechanisms responded to treatment with Na^+^ channel blockers and dantrolene, which lessens Ca^2+^ release from the sarcoplasmic reticulum. Our findings suggest that some hyperKPP patients may benefit from these treatments even if they do not have myotonia detectable by EMG.

To present our findings, we shifted away from the currently used clinical and EMG definitions of myotonia, which are: slowed muscle relaxation following contraction and abnormal spontaneous firing of muscle fiber action potentials in a specific waxing and waning pattern of frequency and amplitude. While these definitions have been useful during clinical evaluations for decades, we found that a more mechanistic definition reflecting the underlying cellular pathophysiology is better able to guide scientific inquiry into the phenotype. We therefore broadly define myotonia as involuntary contraction of muscle fibers that occurs independent of motor axon or neuromuscular junction activation. Within this definition, we identify two distinct types of myotonia in hyperKPP muscle. The first, which we term ‘action potential myotonia’, occurs due to spontaneous firing of muscle fiber action potentials and is readily detected with EMG. The second, which we term ‘non‐action potential myotonia’, is caused by subthreshold membrane depolarizations of muscle fibers that do not trigger action potentials such that it cannot be detected by EMG.

## 2 Materials and Methods

### 2.1 Mice

All animal procedures were performed in accordance with the policies of the Animal Care and Use Committee of Wright State University and were conducted in accordance with the United States Public Health Service's Policy on Humane Care and Use of Laboratory Animals. Mice carrying the M1592V Nav1.4 mutation were used to model hyperKPP (Jackson Labs, FVB.129S4(B6)‐Scn4a<tm1.1Ljh>/J, cat# 011033). HyperKPP mice expressing GCaMP6f in muscle were generated by crossing hyperKPP mice with floxed GCaMP6f mice (Jackson Labs, B6J.Cg‐Gt(ROSA)26Sortm95.1(CAG‐GCaMP6f)Hze/MwarJ, cat #028865) and with parvalbumin promoter driven Cre mice (Jackson Labs, B6.129P2‐Pvalbtm1(cre)Arbr/J, cat# 030218). Mice were sacrificed using CO_2_ inhalation followed by cervical dislocation.

### 2.2 Electrophysiologic Recordings

Isolation of flexor digitorum brevis and interosseous fibers and voltage clamp recordings were performed as previously described [8, 9]. Dissection and preparation of EDL muscles for current clamp recordings were performed as previously described [9]. Solutions containing elevated concentrations of KCl (7 and 11 mM) did not have corresponding reductions in other ions. For experiments performed in 0 extracellular Ca^2+^, 1.5 mM of MgCl_2_ was added to substitute for CaCl_2_ to keep the concentration of divalent cation constant. In addition, 1 mM of EGTA was also added.

### 2.3 Ex Vivo Force Measurements

Force recordings from EDL muscles were performed as previously described [10]. Following determination of the optimal length at 22°C, the temperature of the bath was raised to 35°C, which is close to the in vivo temperature of limb muscles [11]. Once 35°C was reached, the muscle was perfused with normal (3.5 mM) K^+^ solution for 10–20 min, followed by a high K^+^ solution (7 mM or 11 mM) for 45 min, and then returned to normal K^+^ solution for 25 min to follow recovery. 11 mM K^+^ was initially selected as it has been used previously in studies of the mouse model of hyperKPP [7, 12]. To determine the optimum resting length for force generation, the muscle was directly stimulated with two platinum wires placed perpendicular to the EDL muscle. A S‐900 pulse generator (Dagan) provided stimulus pulses. During the recordings, the muscles were not stimulated. All contractions were spontaneous.

### 2.4 Imaging of Ca^2+^ (ΔF/F)

Muscles were visualized without staining using a Leica I3 cube with a bandpass of 450–490 and a longpass of 515, using a 5× objective. A sCMOS camera (CS2100M‐USB) was used to capture images with ThorCam software (Thorlab Inc. NJ). Regions of interest in each muscle type were selected in Image J (NIH). One image was acquired every minute to follow changes in baseline signal. For imaging flashing of fibers during transient myotonia, the camera was switched to continuous mode (30 frames/s). For comparisons of the elevation of baseline Ca2^+^, wild type and hyperKPP EDL muscles expressing GCaMP6f were pinned side‐by‐side and visualized simultaneously.

### 2.5 Statistics

For comparisons between wild type and hyperKPP muscles and for Ca^2+^ imaging, the unpaired Students' *t*‐test was used. Bonferroni correction was applied when multiple comparisons were made between samples. *p* values reported are after Bonferroni correction. For comparison of resting potentials, nested analysis of variance was performed using a script in R. All data is presented as mean ± SD. *p* < 0.05 was considered to be significant.

## 3 Results

**FIGURE 1:**
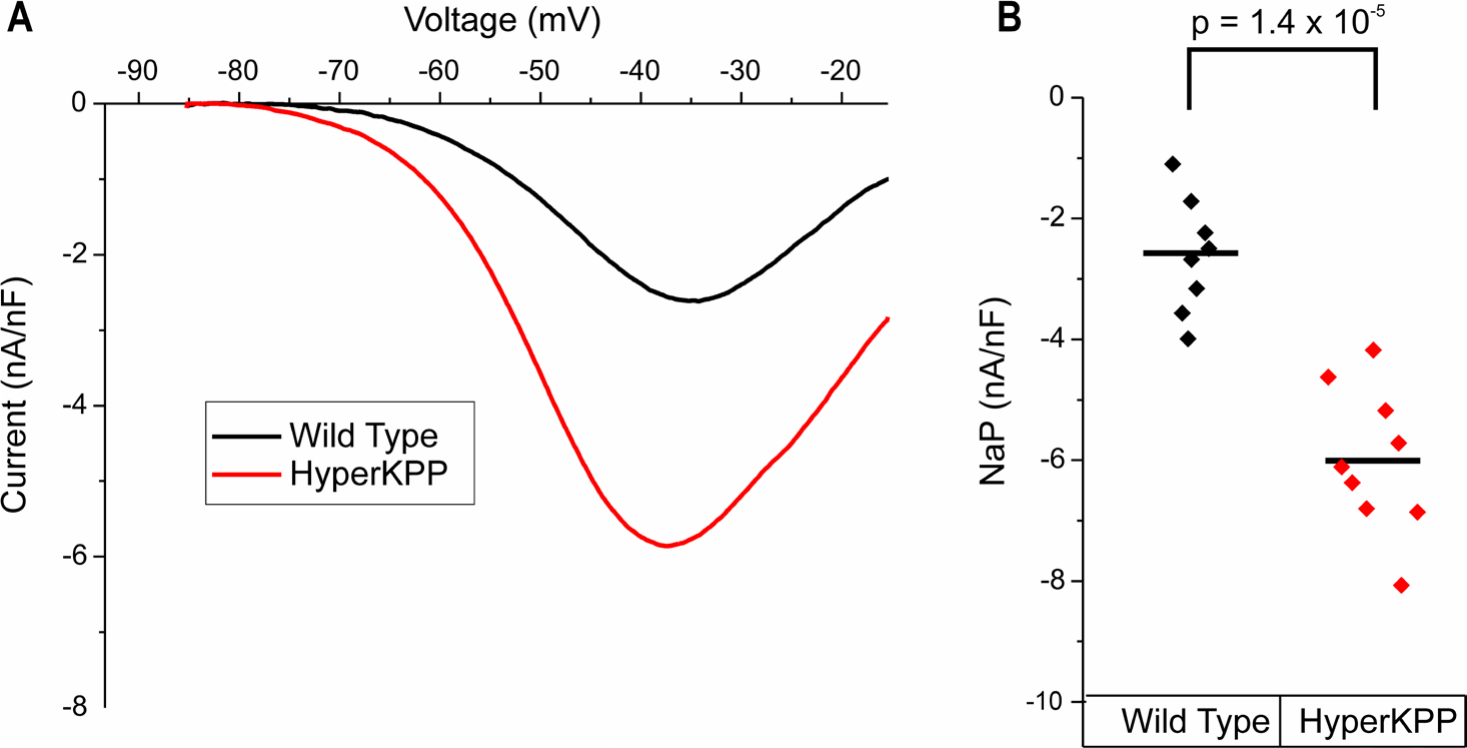
NaP is increased in FDB muscle fibers isolated from hyperKPP mice. (A) Average current–voltage (IV) plot of the NaP in WT (black) and hyperKPP (red). (B) Peak NaP amplitude for WT (*n* = 8 fibers, 3 animals) and hyperKPP (*n* = 9 fibers, 3 animals). The current measurements were normalized to capacitance for each cell. Figure S1 for non‐normalized data.

Voltage clamp recordings were made from FDB and interosseous fibers isolated from both wild type littermates and mice heterozygous for the M1592V mutation in Na_V_1.4 (Figure 1). Ramp depolarizations from −85 to −25 mV were applied over 6 s in solution with blockers of Ca^2+^, Cl,^−^ and K^+^ channels. The slow ramp inactivates the rapidly inactivating Na^+^ current such that the only Na^+^ current activated is NaP [9]. NaP amplitude in hyperKPP fibers was significantly larger than in wild type fibers (Figure 1, 6.0 ± 1.2 nA/uF vs. 2.6 ± 1.0 nA/uF, *p* = 1.4 × 10^−5^).

**FIGURE 2:**
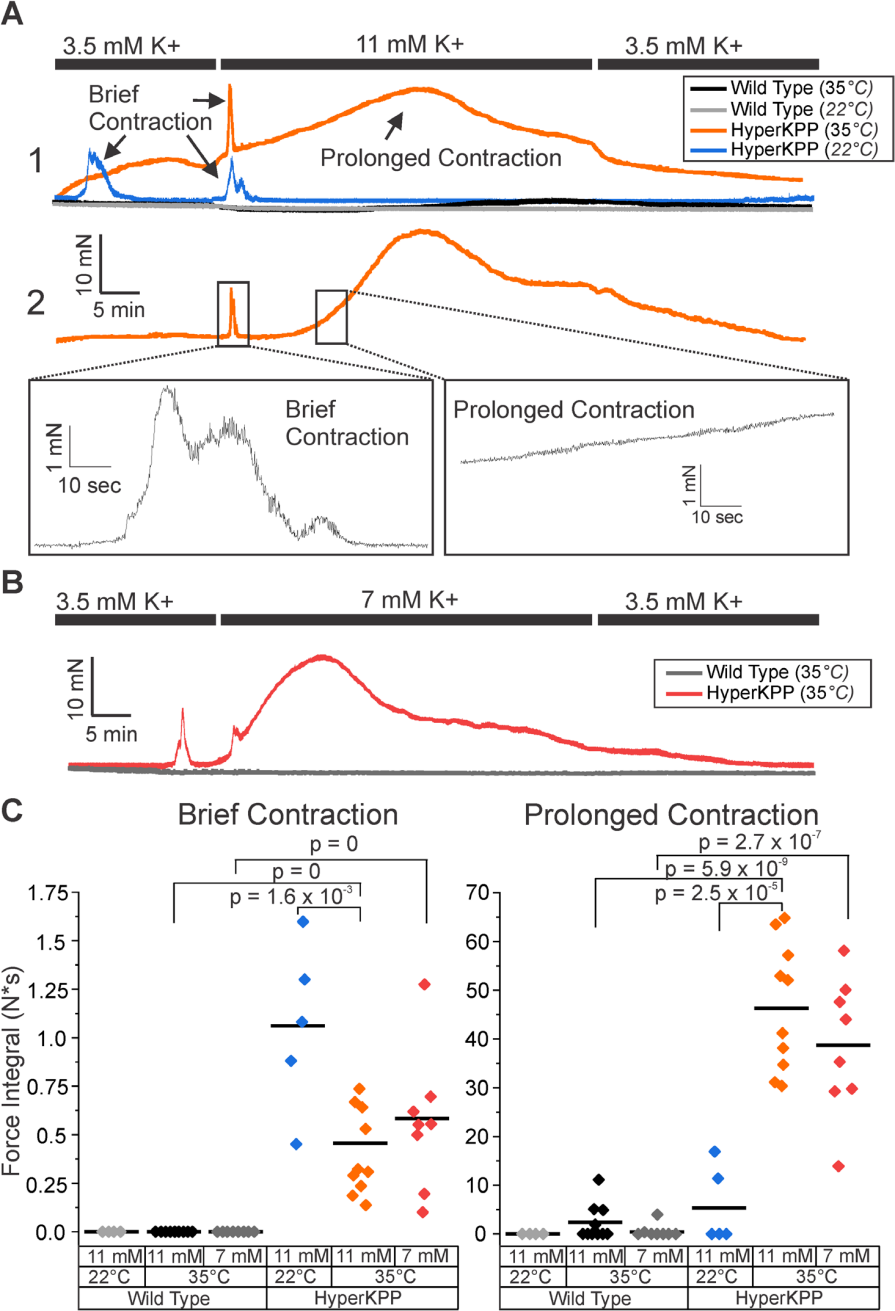
Two distinct mechanisms cause involuntary contraction in hyperKPP muscle. (A1) Four representative traces: HyperKPP at 22°C, hyperKPP at 35°C, WT at 22°C and WT at 35°C. (A2) Expanded time scale of a representative hyperKPP trace displaying the rapidly varying force during brief contraction on the left and the smooth increase in force during prolonged contraction on the right. (B) Representative traces of contraction with perfusion of 7 mM K^+^. (C) Scatter plot of the integral of brief and prolonged contraction with perfusion of high K^+^. For perfusion with 11 mM K^+^ at 22°C, *n* = 5 for hyperKPP and 4 for WT. For 11 mM K^+^ at 35°C, *n* = 10 for both hyperKPP and WT. For perfusion with 7 mM K^+^ at 35°C *n* = 8 for both hyperKPP and WT.

To study involuntary contraction, force recordings were performed ex vivo using the EDL muscle. After dissection, muscles were incubated in 3.5 mM K^+^ for 20 min, followed by elevation of K^+^ to 11 mM for 45 min, with a return to 3.5 mM K^+^ for 25 min. Experiments were performed at both 22°C and 35°C. No involuntary contraction was observed in wild‐type muscles at 22°C. In hyperKPP muscles, at 22°C, contraction lasting less than 5 min was reliably triggered by elevation of K^+^ to 11 mM (Figure 2A). There were also spontaneous bouts of contraction occurring during perfusion of 3.5 mM K^+^ (Figure 2A). At 35°C in hyperKPP muscle, two types of contraction were observed: one brief, which lasted less than 5 min, and one prolonged, which lasted more than an hour (Figure 2A). In wild‐type muscle at 35°C, no brief contraction was observed, but 4/10 muscles did have mild prolonged contraction during perfusion of a solution containing 11 mM K^+^.

Force generated during brief contraction was unstable with rapid fluctuation. In contrast, force generated during prolonged contraction was stable with only gradual increases and decreases (Figure 2A). Prolonged contraction has been observed previously in the mouse model of hyperKPP, but was not characterized [7, 13]. Both brief and prolonged contraction sometimes began prior to elevation of K^+^ (Figure 2A, trace 1). Because serum K^+^ does not reach 11 mM in patients with hyperKPP, we wished to determine whether prolonged contraction could also be triggered by elevation of K^+^ to a level seen in patients. Both brief and prolonged contraction were reliably triggered in hyperKPP muscle by perfusion of 7 mM K^+^, a level seen in patients during attacks of weakness (Figure 2B) [14, 15].

Brief and prolonged contraction were quantitated by taking the integral of force with respect to time to include consideration of both severity and duration (Figure 2C). At 35°C, every hyperKPP muscle had prolonged contraction, which was responsible for close to 99% of contraction (*n* = 18, Figure 2C). There were no differences in the severity of prolonged contraction in hyperKPP muscle when triggered by perfusion with 7 mM K^+^ or 11 mM K^+^ (Figure 2C). In hyperKPP muscle, brief contraction was more severe at 22°C, whereas prolonged contraction was more severe at 35°C (Figure 2C). The opposite temperature dependence indicates brief and prolonged contraction are caused by distinct mechanisms.

**FIGURE 3:**
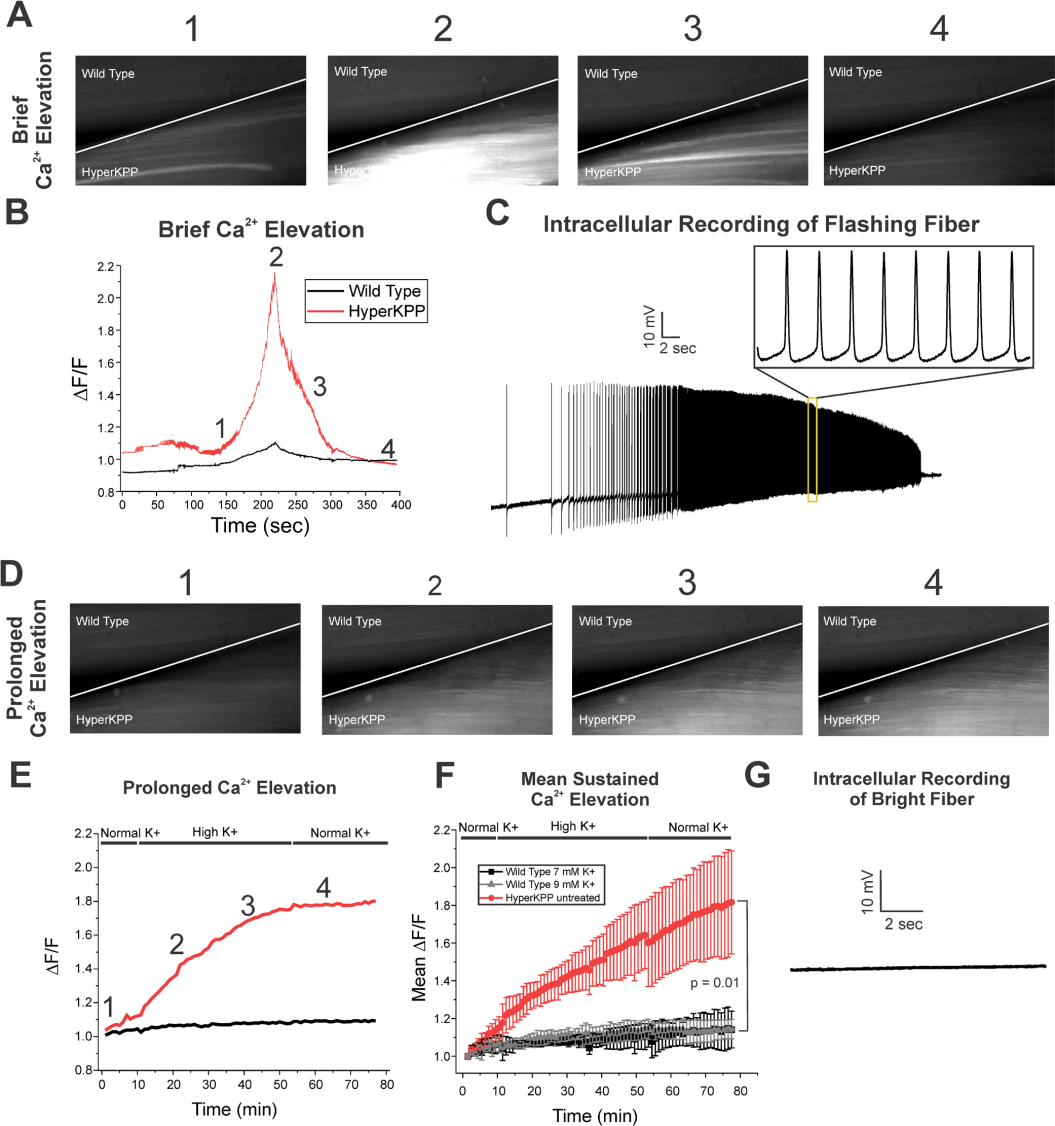
Brief contraction is due to action potentials whereas prolonged contraction occurs in the absence of action potentials. (A) A pair of WT and hyperKPP muscles imaged over a 5‐min period during flashing of hyperKPP fibers caused by elevation of K^+^ to 7 mM. (B) Plot of ΔF/F for the WT and HPP muscles shown in A. Pictures 1–4 relate to timepoints 1–4 on the graph. (C) Intracellular recording from a flashing fiber showing a myotonic discharge. (D) Images of a WT and a hyperKPP muscle taken over 80 min during perfusion with 7 mM K^+^. (E) Plot of ΔF/F for the WT and hyperKPP muscles shown in D. (F) The mean elevation of ΔF/F for WT and hyperKPP in 7 mM K^+^ (*p* = 0.01, *n* = 4 muscles for each group). Error bars represent SD. (G) Intracellular recording from a fiber during the sustained elevation of ΔF/F. There was no firing of action potentials in 18 fibers from 3 mice.

Contraction of skeletal muscle is triggered by elevation of intracellular Ca^2+^ [16, 17]. To determine whether elevation of intracellular Ca^2+^ was the mechanism underlying prolonged contraction, we crossed hyperKPP mice with mice expressing GCAMP6f in skeletal muscle. GCAMP6f is a high‐affinity Ca^2+^ indicator with a Kd near 600 nM such that it can detect changes in signal (ΔF/F) at relatively low intracellular Ca^2+^ concentrations [18, 19]. Contraction was blocked by preincubation in N‐benzyl‐p‐toluenesulfonamide (BTS) [20]. Imaging was performed prior to, during, and following perfusion with a solution containing 7 mM K^+^ at 35°C. For comparison, in each experiment, one wild‐type muscle and one hyperKPP muscle expressing GCAM6f were placed side by side and imaged simultaneously. No significant increase in Ca^2+^ signal was observed in wild‐type muscles (Figure 3A,D). In contrast, in hyperKPP muscle, infusion of 7 mM K^+^ triggered flashing of fibers suggesting myotonic discharges (Figure 3A, Video S1). The elevation of mean whole muscle Ca^2+^ signal due to flashing of individual fibers lasted 1–5 min (Figure 3B), corresponding to brief contraction. To determine whether flashing of fibers was due to myotonic discharges, we impaled fibers that were flashing. This was challenging as fibers flashed only briefly and only surface fibers could be impaled. Two flashing fibers were successfully impaled while they flashed and myotonic discharges were present in both (Figure 3C).

In addition to the flashing of individual fibers, in hyperKPP muscles, there was a prolonged elevation of Ca^2+^ that involved all fibers and evolved over many minutes (Figure 3D–F, Video S2). The prolonged elevation in Ca^2+^ persisted following the return of extracellular K^+^ to 3.5 mM (Figure 3D–F). When the intensity of the Ca^2+^ signal at the end of imaging (following return to normal K^+^) in hyperKPP was compared to wild type, there was a significant difference (Figure 3F). During the prolonged elevation of Ca^2+^, no action potentials were observed in 18 fibers impaled from 3 hyperKPP muscles in a solution containing 7 mM K^+^ (Figure 3G). We conclude brief elevations of Ca^2+^ are due to myotonic discharges and thus can be categorized as action potential myotonia (AP myotonia) whereas sustained elevation of Ca^2+^ occurs in fibers not firing action potentials (non‐AP myotonia).

**FIGURE 4:**
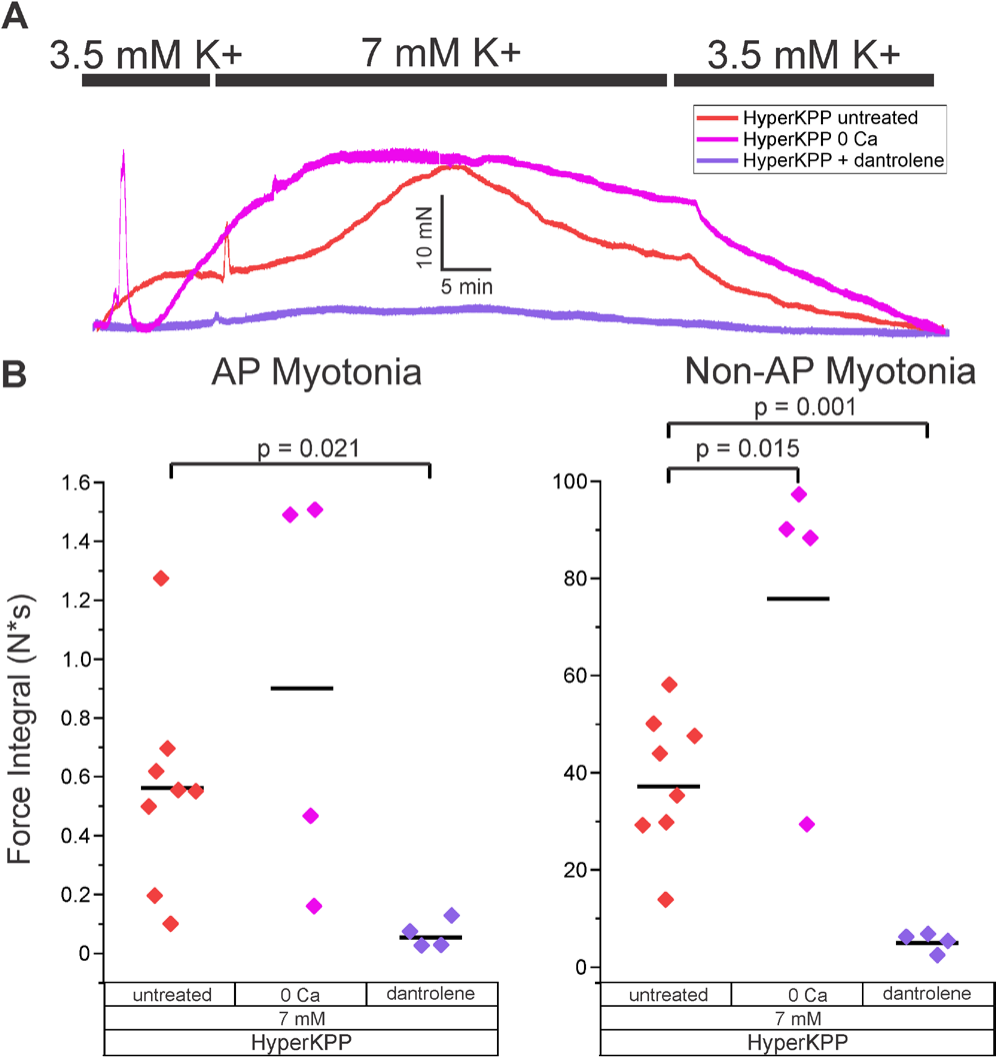
Ca^2+^ release from the sarcoplasmic reticulum is responsible for both AP myotonia and non‐AP myotonia. (A) Representative traces from hyperKPP untreated, hyperKPP exposed to 20 μM dantrolene and hyperKPP in 0 extracellular Ca^2+^. (B) Integral of both AP myotonia and non‐AP myotonia for hyperKPP in 0 Ca^2+^ (*n* = 4 muscles) and hyperKPP in a solution containing 20 μM dantrolene (*n* = 4). Data for untreated hyperKPP muscles from Figure 2C is plotted to allow for comparison.

Cytoplasmic Ca^2+^ can be elevated by Ca^2+^ coming from either extracellular or intracellular compartments. Reduction in extracellular Ca^2+^ by not adding Ca^2+^ to the extracellular solution and instead adding 1 mM EGTA caused a significant increase in non‐AP myotonia, suggesting the Ca^2+^ triggering non‐AP myotonia was coming from an intracellular store (Figure 4A,B, *n* = 4). The normal trigger for contraction of skeletal muscle is Ca^2+^ release from the sarcoplasmic reticulum via the ryanodine receptor (RYR1) [21]. Dantrolene is a blocker of RYR1 and is used to reduce Ca^2+^ release from the sarcoplasmic reticulum during attacks of malignant hyperthermia [22, 23, 24]. 20 μM dantrolene reduced AP myotonia by 89% ± 73.9% (Figure 4A,B, *n* = 4). This was expected since the mechanism underlying action potential‐induced contraction is the release of Ca^2+^ from the sarcoplasmic reticulum. Non‐AP myotonia was reduced to a similar degree (86% ± 36.9%, Figure 4). These data suggest that Ca^2+^ triggering non‐AP myotonia is released from the sarcoplasmic reticulum via a mechanism independent of action potentials.

**FIGURE 5:**
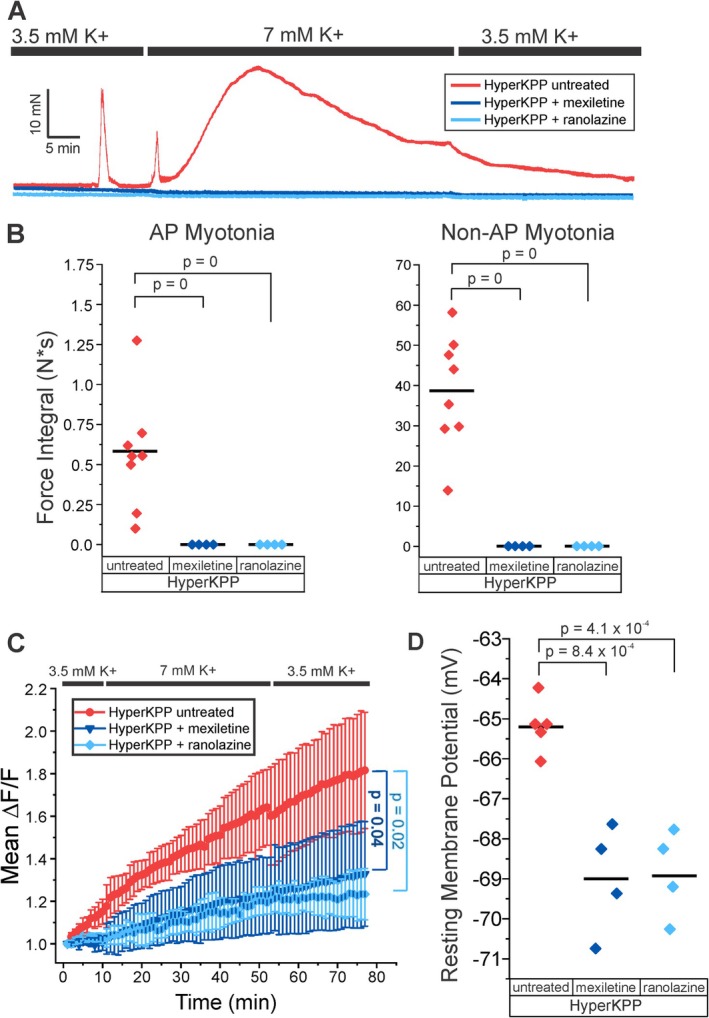
Na^+^ channel blockers eliminate AP myotonia, non‐AP myotonia and lessen elevation of intracellular Ca. (A) Representative traces from untreated hyperKPP and following treatment with 20 μM mexiletine or 40 μM ranolazine. (B) Scatter plots of the integral of both AP myotonia and non‐AP myotonia for mexiletine treated (*n* = 4, dark blue) and ranolazine treated hyperKPP (*n* = 4, light blue). Data from Figure 2 for untreated hyperKPP is shown for comparison. (C) The mean Ca^2+^ elevation for mexiletine treated (*n* = 4, dark blue) and ranolazine treated hyperKPP (*n* = 3, light blue). The mean intracellular elevation of Ca^2+^ levels in untreated hyperKPP presented in Figure 3F is shown for comparison. (D) Scatter plot of mean resting membrane potential for hyperKPP muscles in 7 mM K^+^. Each point represents the mean of at least 6 fibers from each muscle.

Mexiletine is the current standard of care in treating myotonia in muscle diseases, but ranolazine is also effective [25, 26]. Both Na^+^ channel blockers are known to be effective in lessening myotonic discharges. However, since non‐AP myotonia occurred in fibers not firing action potentials, it was unclear whether Na^+^ channel blockers would be effective as treatment. HyperKPP muscles were treated with 20 uM mexiletine or 40 uM ranolazine. Both Na^+^ channel blockers significantly reduced both AP myotonia and non‐AP myotonia (Figure 5A,B). To determine the mechanism underlying the efficacy of both drugs, muscle Ca^2+^ was imaged during the infusion of 7 mM K^+^ in hyperKPP mice expressing GCAMP6f in muscle. Both ranolazine and mexiletine significantly reduced the prolonged elevation of intracellular Ca^2+^ (Figure 5C). To confirm that both drugs were blocking NaP in hyperKPP muscle, resting potential was compared between untreated and treated muscle. Both drugs significantly lessened depolarization of hyperKPP muscle following exposure to 7 mM K^+^ (Figure 5D). These data strongly suggest that activation of NaP, via depolarization, Na^+^ overload, or both, triggers the prolonged elevation of intracellular Ca^2+^ that occurs during non‐AP myotonia.

**FIGURE 6:**
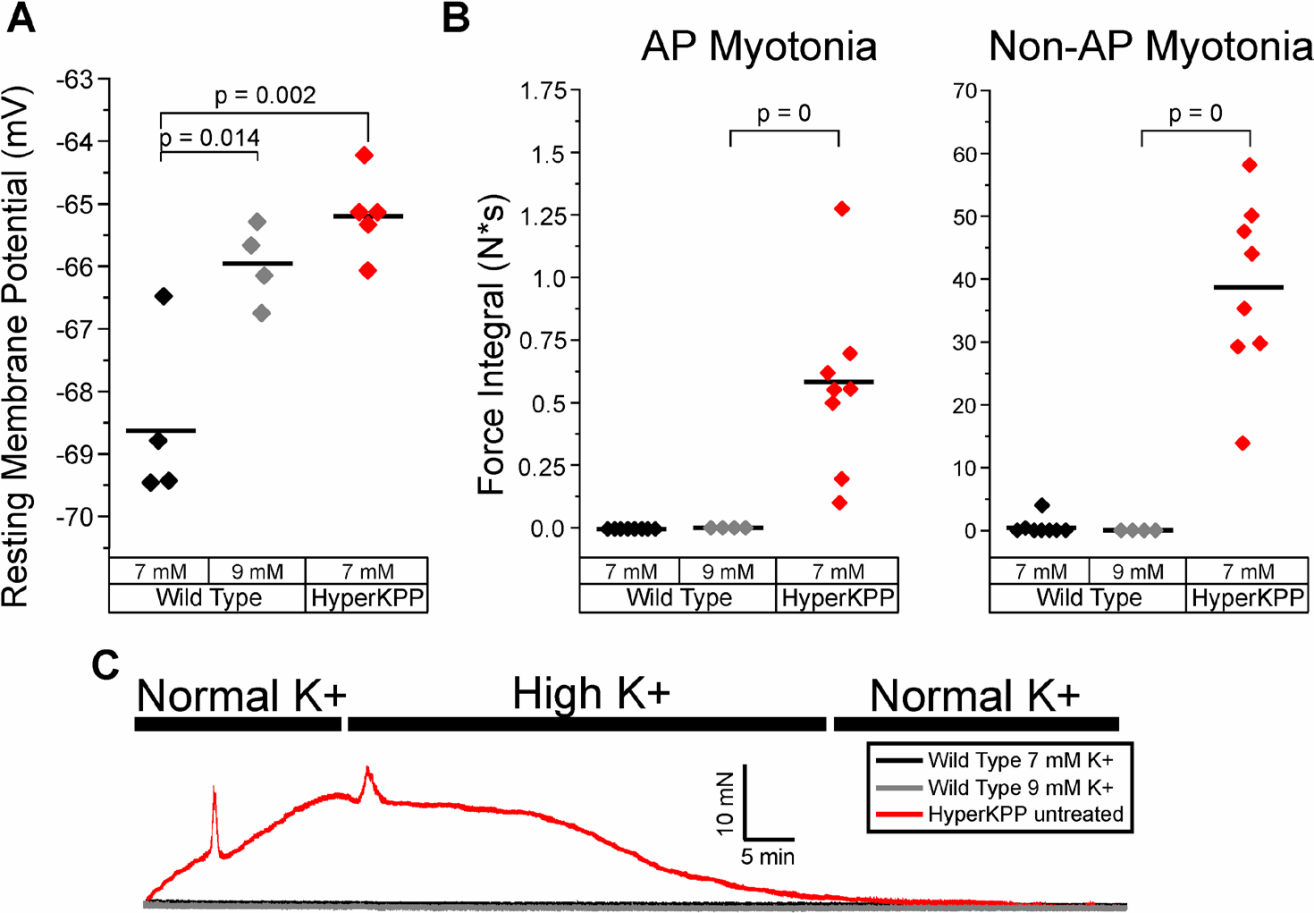
Depolarization of the resting membrane potential (RMP) in hyperKPP is not sufficient to trigger either AP myotonia or non‐AP myotonia. (A) Scatter plot of mean muscle RMPs in WT in 7 mM K^+^ at 35°C (black, *n* = 4 muscles), WT in 9 mM K^+^ (gray, *n* = 4 muscles). Data for hyperKPP muscle in 7 mM K^+^ from Figure 5D is shown for comparison. (B) Integral of both AP myotonia and non‐AP myotonia for WT muscle in 9 mM K^+^ at 35°C. Data from Figure 2C for WT in 7 mM K^+^ and hyperKPP in 7 mM are re‐plotted to allow for comparison. (C) Representative force traces with perfusion of 7 and 9 mM K^+^.

One possibility is that elevation of intracellular Ca^2+^ causing non‐AP myotonia is entirely explained by depolarization of the resting potential. If this were the case, depolarization of wild type muscle to the same degree as hyperKPP muscle in 7 mM K^+^ should trigger non‐AP myotonia. In agreement with a previous study, the resting potential of wild type muscle in 7 mM K^+^ was 3.3 mV less depolarized than hyperKPP muscle (−68.5 ± 1.4 mV in WT vs. −65.2 ± 0.7 mV in hyperKPP, *p* = 0.014) [12] (Figure 6A). To depolarize WT muscle to the same resting potential as hyperKPP muscle in 7 mM K,^+^ we raised K^+^ to 9 mM, which depolarized the membrane potential to −66.0 ± 0.6 mV (Figure 6A, *p* = 0.11 vs. hyperKPP in 7 mM K^+^). Depolarization of WT muscle to −66 mV did not trigger either AP myotonia (*p* = 1 vs. WT in 7 mM K^+^, *n* = 8 WT and 4 hyperKPP) or non‐AP myotonia (*p* = 0.46, Figure 6B,C). Furthermore, elevation of K^+^ to 9 mM did not trigger a significant elevation of intracellular Ca^2+^ (*p* = 0.99 vs. WT in 7 mM K^+^, *n* = 4). These data demonstrate that a mechanism distinct from depolarization contributes to elevation of intracellular Ca^2+^ and generation of non‐AP myotonia in hyperKPP muscle.

## 4 Discussion

Current understanding of myotonia in muscle ion channelopathies is that it is due to a single mechanism: involuntary firing of myogenic action potentials (myotonic discharges) that are readily detectable by EMG. However, in studies of a mouse model of hyperKPP, we identified two distinct mechanisms that contribute to involuntary contraction. The first, which we term AP myotonia, is caused by myotonic discharges and corresponds to the prevailing clinical definition. The second, which we term non‐AP myotonia, is caused by action potential‐independent release of Ca^2+^ from the sarcoplasmic reticulum. Both Na^+^ channel blockers and inhibition of Ca^2+^ release from the sarcoplasmic reticulum by dantrolene were effective in treating both causes of myotonia.

### 4.1 AP Myotonia in hyperKPP

Approximately 50% of hyperKPP patients report having myotonia [27]. The AP myotonia detected in the current study corresponds to myotonia in hyperKPP patients that can be detected by EMG. AP myotonia occurred spontaneously (without either prior contraction or stretch) or with elevation of extracellular K^+^ and was more severe at lower temperatures, suggesting it might contribute to the worsening of myotonia with cooling in the paramyotonia congenita phenotype experienced by a subset of patients with hyperKPP Scn4a mutations [27].

### 4.2 Non‐AP Myotonia in hyperKPP

In the current study, prolonged contraction of muscle occurred in the absence of action potentials. Two findings support the conclusion that non‐AP myotonia in hyperKPP muscle is due to an action potential‐independent elevation of intracellular Ca^2+^. (1) Fibers impaled during the sustained increase in Ca^2+^ were not firing myotonic discharges. (2) Treatments that lessened non‐AP myotonia also lessened the sustained elevation of intracellular Ca^2+^. However, there was a discrepancy between the duration of elevation of intracellular Ca^2+^ and the duration of non‐AP myotonia. Non‐AP myotonia decreased in severity after 1 h. In contrast, the elevation of intracellular Ca^2+^ persisted. A potential explanation for this discrepancy is fatigue.

The search for non‐AP myotonia in hyperKPP was inspired by our recent discovery of plateau potentials in myotonia congenita. Plateau potentials are depolarizations lasting seconds to minutes, during which intracellular Ca^2+^ can be elevated, despite inexcitability of muscle [28, 29]. Two findings suggest the mechanism underlying action potential independent elevation of Ca^2+^ may differ between hyperKPP and myotonia congenita. The first is that the duration of the elevation in Ca^2+^ differs: in myotonia congenita it lasted seconds to a few minutes whereas in hyperKPP it lasted for more than 1 h. Second, the elevation of Ca^2+^ in myotonia congenita occurred during depolarization of muscle to near −30 mV, whereas in hyperKPP, the elevation occurred in muscle that was only depolarized to near −65 mV. In wild type muscle, contraction triggered by elevation of extracellular K^+^ begins when the membrane potential reaches −40 mV [30]. These data suggest the depolarization in myotonia congenita is sufficient to trigger elevation of Ca^2+^ whereas depolarization of hyperKPP fibers is insufficient. Further evidence that depolarization alone is insufficient came from the finding that depolarization of wild type muscle to the same membrane potential as hyperkPP muscle in 7 mM K^+^ did not trigger either elevation of Ca^2+^ or myotonia. Additionally, non‐AP myotonia often began prior to elevation of extracellular K^+^, when the resting potential would not be significantly depolarized. Taken together, these findings suggest depolarization is not the sole mechanism triggering non‐AP myotonia in hyperKPP.

**FIGURE 7:**
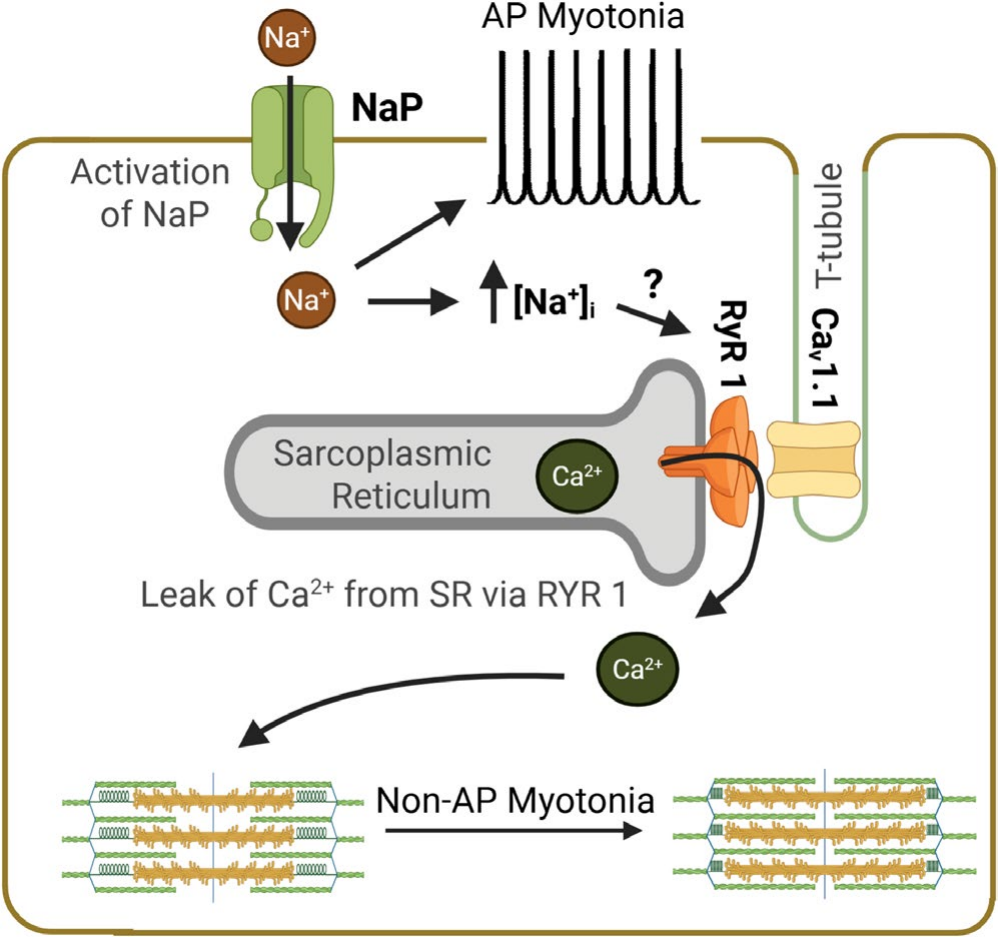
The sequence of events leading to AP myotonia and non‐AP myotonia. Activation of NaP, via depolarization of the membrane potential, acutely triggers myotonic discharges, which underlie AP myotonia. Chronic activation of NaP causes Na^+^ build‐up in muscle, which via an unknown mechanism, causes Ca^2+^ leak from the sarcoplasmic reticulum via RYR1 and non‐AP myotonia.

We propose activation of persistent Na^+^ current (NaP) triggers myotonia by the pathways illustrated in Figure 7. In the schema, activation of NaP directly triggers AP myotonia via suprathreshold depolarization of the membrane potential [31]. Over time, subthreshold membrane depolarization causes chronic activation of NaP that, in turn, causes Na^+^ overload, triggering sustained elevation in intracellular Ca^2+^ and non‐AP myotonia.

Several findings support the possibility that Na^+^ overload triggers non‐AP myotonia. The first is that treatment with the Na^+^ channel blockers mexiletine and ranolazine, which are thought to preferentially block NaP [32, 33], greatly reduced non‐AP myotonia. At the resting potential of hyperKPP muscle in 7 mM K^+^, NaP is chronically active, as evidenced by the 3 mV of extra depolarization of hyperKPP muscle. Both Na^+^ channel blockers eliminated the difference in resting potential between hyperKPP and WT muscle, strongly suggesting they block chronic activation of NaP at the concentrations used. Consistent with the possibility of Na^+^ overload is the finding that hyperKPP patients have increased intracellular Na^+^ in muscle during attacks of weakness [34].

The source of Ca^2+^ responsible for non‐AP myotonia appears to be the sarcoplasmic reticulum (SR). Dantrolene, a ryanodine receptor (RYR1) inhibitor, prevents Ca^2+^ release from the SR in malignant hyperthermia [23, 35, 36] and greatly reduces non‐AP myotonia. The link between elevation of intracellular Na^+^ and leak of Ca^2+^ from the SR via RYR1 is unknown. In the heart, block of Na^+^ channels can reverse detrimental effects of Ca^2+^ leak through RYR2 channels in catecholaminergic polymorphic ventricular tachycardia [37, 38]. Although the mechanism is unclear, it has been proposed that there is cross talk between Na^+^ channels and RYR receptors [38, 39]. The finding that block of Na^+^ channels can prevent non‐AP myotonia parallels findings in the heart and suggests there may be similar cross talk in skeletal muscle.

### 4.3 Treatment of HyperKPP

Only a minority of hyperKPP patients are treated with Na^+^ channel blockers [27]. One reason for this may be reports that Na^+^ channel blockers are ineffective at preventing attacks of weakness [40, 41]. While complaints of myotonia are not prominent in hyperKPP, approximately 50% of patients experience pain and fatigue [42]. It is possible that muscle pain and fatigue are caused by non‐AP myotonia. Our discovery of the responsiveness of non‐AP myotonia to Na^+^ channel blockers suggests that channel blockers may benefit hyperKPP patients even if they lack both clinical and EMG evidence of myotonia.

Approximately one third of patients with hyperKPP experience progressive myopathy as they age [27, 43, 44]. The cause of myopathy is unknown. Our discovery of prolonged elevation of intracellular Ca^2+^ during non‐AP myotonia provides a potential mechanism. Prolonged elevation of intracellular Ca^2+^ can trigger cell death via apoptosis, necroptosis, and ferroptosis [45, 46, 47]. The finding that Na^+^ channel blockers prevent sustained elevation of Ca^2+^ due to activation of NaP raises the possibility that they might lessen severity or slow progression of the chronic myopathy.

Our data suggest a potentially novel approach to therapy of muscle pain and fatigue in hyperKPP patients who cannot tolerate Na^+^ channel blockers. Dantrolene was effective in treating non‐AP myotonia in our ex vivo preparation. Whether hyperKPP patients in which non‐AP myotonia is a prominent contributor to muscle dysfunction would benefit from treatment with dantrolene is an area for further study. Of note, dantrolene has been given to patients with Duchenne muscular dystrophy, was well tolerated, and did not cause worsening of weakness due to disruption of excitation contraction coupling [48].

### 4.4 A Revised Definition of Myotonia

Myotonia is defined clinically as slowed muscle relaxation following contraction or electrically, by EMG, as abnormal spontaneous firing of muscle fiber action potentials in a specific waxing and waning pattern of frequency and amplitude. Despite their clinical utility and ubiquitous use, these definitions bypass discussion of underlying cellular pathophysiology and, in doing so, may hinder development of novel, targeted therapeutics. Therefore, in the current study we revise the definition of myotonia to a transient involuntary muscle contraction that occurs independent of motor axon or neuromuscular junction activation (i.e., originating in the myofibers themselves). We further subdivide this definition, as noted above, into contractions occurring due to myotonic discharges (AP myotonia) and those occurring in the absence of action potentials (non‐AP myotonia).

The key aspect of the new definition is that myotonia is a transient, involuntary contraction of muscle that originates in muscle and is due to pathology of muscle membrane ion channels. This definition includes both AP and non‐AP myotonia. Whereas AP myotonia is underlain by spontaneous suprathreshold membrane depolarizations that trigger repetitive firing of action potentials that wax and wane in frequency, non‐AP myotonia is underlain by spontaneous subthreshold membrane depolarizations that result in prolonged release of Ca^2+^ from the sarcoplasmic reticulum in the absence of action potentials. Though both AP myotonia and non‐AP myotonia are electrical phenomena, non‐AP myotonia would be missed by EMG, which can only pick up large, rapid changes in muscle membrane potential. Non‐AP myotonia can only be identified and studied using advanced imaging and intracellular recording techniques, which are much more sensitive to small changes in membrane potential. These techniques are required to identify new cellular targets and, by extension, treatment strategies for both AP and non‐AP myotonia.

The current clinical and EMG definitions can become unwieldy when used to discuss studies of mechanisms underlying myotonia. For example, defining clinical myotonia as delayed relaxation following voluntary contraction limits the discussion of spontaneous or percussion‐induced contractions observed in myotonic disorders. In contrast, AP myotonia encompasses all involuntary contractions caused by myogenic action potentials, which is a more expansive clinical definition but maintains a common underlying pathophysiology. Similarly, defining electrical myotonia by waxing and waning discharges on EMG limits discussion on involuntary contractions that are due to subthreshold depolarizations and are, therefore, electrically silent on EMG. While the term contracture is typically used to discuss muscle contraction occurring in the absence of action potentials on EMG, including in the muscle ion channelopathy paramyotonia congenita [49, 50], the definition of contracture is unclear and includes the permanent shortening of muscle due to fibrosis [51]. In contrast, non‐AP myotonia provides a mechanistic definition for transient muscle contraction that is electrically silent on EMG and is distinct from fibrosis. It should be noted that rigor mortis or loss of Ca^2+^ homeostasis due to a generalized metabolic problem such as hypoxemia in the middle of the EDL muscle could cause muscle contraction in the absence of action potentials. However, non‐AP myotonia occurs in healthy, living cells as demonstrated with intracellular recording showing that fibers with sustained elevation of Ca^2+^ had resting potentials near −65 mV and were able to fire action potentials. Furthermore, resting potentials recovered to normal following return to solution containing normal extracellular K^+^ (data not shown). Finally, Na^+^ channel blockers prevented non‐AP myotonia. They would not be expected to prevent either metabolic problems due to hypoxia or death of fibers. We conclude non‐AP myotonia is not caused by nonspecific metabolic compromise or rigor mortis.

Applying our definition results in myotonia being a heterogenous phenotype, but promotes more specific discussion of underlying mechanisms. We chose the terms AP myotonia and non‐AP myotonia as this seemed an obvious first step in categorizing causes of myotonia but expect (as we have seen in myotonia congenita and discuss further below) there are likely several mechanisms that trigger non‐AP myotonia. Further descriptors could be applied to describe the various mechanisms causing myotonia.

### 4.5 Contribution of Non‐AP Myotonia to Muscle Dysfunction in Other Diseases

Non‐AP myotonia has been previously described in several muscle diseases. The closest to hyperKPP is paramyotonia congenita in which Nav1.4 mutations cause cold‐induced non‐AP myotonia [49]. Another is malignant hyperthermia in which Ca^2+^ release from the SR due to mutations of the ryanodine receptor causes life‐threatening non‐AP myotonia triggered by volatile anesthetics [52]. Prominent non‐AP myotonia occurs in Brody myopathy due to loss of function of the ATP2A1 gene (SERCA1 protein) with resultant slowed uptake of Ca^2+^ into the sarcoplasmic reticulum and slowed relaxation following high‐intensity exercise, which is worsened by cold [53, 54]. Non‐AP myotonia can also be found in McArdle disease (glycogen storage disease type V) although the non‐AP myotonia is associated with a number of other findings such as rhabdomyolysis and exercise intolerance [55].

It is also intriguing, yet speculative, to consider whether non‐AP myotonia may be responsible for symptoms in patients suffering from unexplained muscle stiffness and pain. Myalgia is a common complaint encountered by neuromuscular and primary care physicians [56], but is rarely proven attributable to underlying muscle pathology. However, myotonic dystrophy type 2 (DM2) may present with early, severe, painful cramps or myalgia in the absence of clinical or EMG myotonia and is often indistinguishable from fibromyalgia [57, 58, 59, 60]. Similarly, at least one gain of function mutation in the SCN4A gene is associated with myalgia and muscle stiffness in the absence of clinical myotonia [61], while others may cause painful cramps in addition to myotonia [62, 63, 64, 65]. Whether or not NaP current is affected in any of these conditions is unknown, but many cases of unexplained muscle pain are treatment resistant, which suggests that conventional treatments may not match the underlying pathophysiology. Moreover, population‐based studies suggest the prevalence of persistent muscle pain to be approximately 10% or more [56, 66]. Together, these demonstrate a clear need for well‐defined, mechanistic understandings of muscle pain. If non‐AP myotonia is contributory in a subset of cases, sodium channel blockers or dantrolene may have benefit.

A subset of wild type muscles had mild non‐AP myotonia. NaP is present in wild type muscle, albeit to a lesser degree than in hyperKPP muscle (Figure 1). It is possible that there is Na^+^ overload and Ca^2+^ leak from the SR in some wild type muscles exposed to high K^+^. We studied young mice; Ca^2+^ leak from the SR has been shown to increase with aging [67] as does muscle stiffness [68]. Future studies will be needed to determine whether non‐AP myotonia contributes to muscle dysfunction in aging.

## 5 Conclusion

We demonstrated in a mouse model of hyperKPP that myotonia is not a single phenomenon, but is rather a heterogeneous phenotype caused by distinct mechanisms including both suprathreshold and subthreshold membrane depolarizations, which we term AP and non‐AP myotonia, respectively. Our data further suggest that patients with hyperKPP who suffer from muscle pain and stiffness may benefit from Na^+^ channel blockers even if they have no clinical or EMG evidence of myotonia, as those symptoms may be caused by non‐AP myotonia. Moreover, because Na^+^ channel blockers treat the underlying Na^+^ channel gain of function and reduce chronic subthreshold depolarizations, they may prevent episodes of pathologic elevation of intracellular Ca, which could slow progression to chronic myopathy. Further study is, of course, necessary and warranted.

## Author Contributions

C.D., A.D., and M.N. all participated in the acquisition and analysis of data. C.D., A.D., A.A.V., and M.M.R. were involved in the conception and design, as well as drafting a significant portion of the manuscript.

## Conflicts of Interest

The authors declare no conflicts of interest.

## Supporting information


**Data S1:** AP myotonia in HyperKPP muscle.

**Data S2:** Non‐AP myotonia in HyperKPP muscle.

**Figure S1:** NaP is increased in FDB muscle fibers isolated from hyperKPP mice. (A) Average current–voltage (IV) plot of the NaP in WT (black) and hyperKPP (red). (B) Peak NaP amplitude for WT (*n* = 8 fibers, 3 animals) and hyperKPP (*n* = 9 fibers, 3 animals).

## Data Availability

Data will be made available upon request from the corresponding author.
