# Supplementary figures and images for: Discovery and Treatment of Action Potential‐Independent Myotonia in Hyperkalemic Periodic Paralysis
Source: Ann Clin Transl Neurol. 2025 Jul 14;12(10):2056–67. doi: 10.1002/acn3.70134 (PMC12516254; doi:10.1002/acn3.70134)

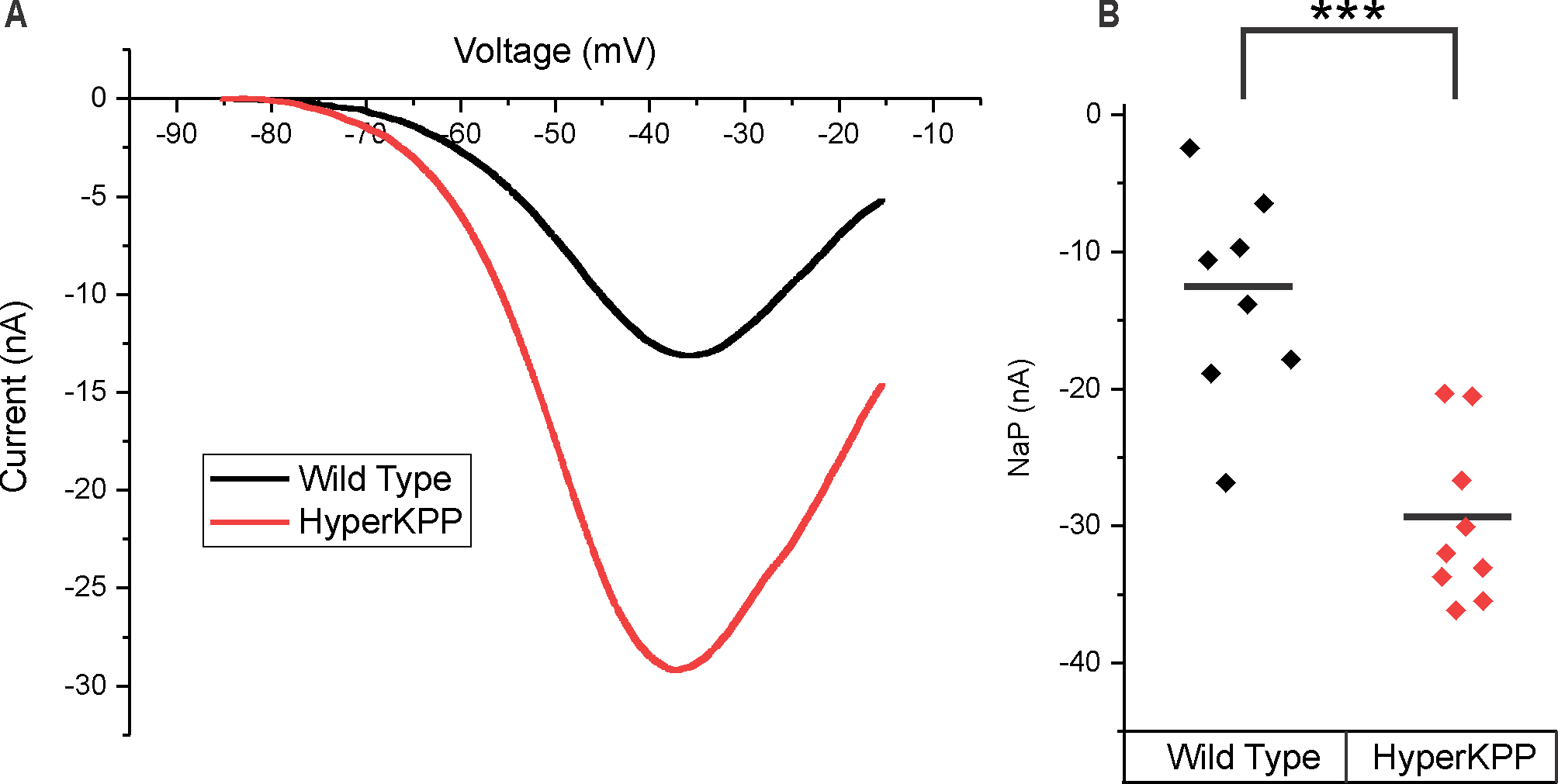

Supplement: Supplementary file 3 — Figure S1: NaP is increased in FDB muscle fibers isolated from hyperKPP mice. (A) Average current–voltage (IV) plot of the NaP in WT (black) and hyperKPP (red). (B) Peak NaP amplitude for WT (n = 8 fibers, 3 animals) and hyperKPP (n = 9 fibers, 3 animals). [file ACN3-12-2056-s002.tif]
